# Mental Health in Postoperative Thyroid Patients During the COVID-19 Pandemic

**DOI:** 10.3389/fendo.2022.875325

**Published:** 2022-06-28

**Authors:** Shijie Yang, Xiequn Xu

**Affiliations:** Department of General Surgery, Peking Union Medical College Hospital, Chinese Academy of Medical Sciences and Peking Union Medical College, Beijing, China

**Keywords:** thyroid surgery, mental health, COVID-19, anxiety, depression

## Abstract

**Background:**

Little is known about mental health in patients after thyroid surgery during the peak of the COVID-19 pandemic in China. This study aimed to assess the mental health of postoperative thyroid patients and to explore potential factors associated with psychological symptoms.

**Methods:**

In this study, we surveyed 241 patients who underwent thyroid surgery at Peking Union Medical College Hospital. Insomnia, anxiety, depression, and posttraumatic stress symptoms (PTSS) were measured using the Insomnia Severity Index (ISI), Generalized Anxiety Disorder Questionnaire (GAD-7), Patient Health Questionnaire (PHQ-9), and Impact of Event Scale-Revised (IES-R), respectively.

**Results:**

A significant proportion of postoperative patients reported experiencing insomnia, anxiety, depression, and PTSS. Patients that were older, single/divorced/widowed, and less educated; had lower income and poor general health; had undergone surgery within the past six months; had disrupted follow-up, and; searched social media for COVID-19-related information were associated with worse mental health.

**Conclusions:**

During the COVID-19 pandemic, postoperative thyroid patients tended to develop mental health problems and have less psychological support, emphasizing the importance of patient education and psychological interventions.

## Introduction

With the coronavirus disease 2019 (COVID-19) pandemic outbreak at the end of 2019, the disease rapidly spread worldwide. In the initial period, China was the most impacted by this pandemic, and there was an accompanying epidemic of anxiety and depression nationwide (1–3). People experienced numerous inconveniences due to the implementation of home quarantine policies (4, 5). Additionally, hospitals admitted large numbers of COVID-19 patients, and the shortage of frontline medical staff resulted in limited follow-up for postoperative patients (6, 7). Postoperative thyroid patients require close monitoring and treatment, and the majority of these patients are also cancer patients, who may be more vulnerable to negative emotions that could lead to psychological problems (8–10).

Several studies have explored anxiety, depression, and quality of life during the COVID-19 pandemic in a variety of populations, including the general population (1, 2, 11), health care workers (12–14), medical students (15, 16), cancer patients (17–19), and postoperative surgical patients (20, 21). The majority of these study populations experienced negative emotional states during the pandemic; however, to date, no studies have focused on the mental health of patients after thyroid surgery (22). While thyroid cancer is one of the more inert cancers with a relatively good prognosis, a diagnosis of thyroid nodules, especially thyroid cancer, would affect a patient’s postoperative psychological status. And several studies have reported similar or greater mental burden in thyroid patients (23, 24). The prevalence of thyroid cancer has increased every year, as has the volume of thyroid surgery (25–27), and the quality of life for postoperative thyroid patients has been of great concern to researchers (28, 29). However, during the COVID-19 pandemic, postoperative thyroid patients did not receive attention commensurate with their numbers.

The current study employed 4 validated psychological scales to assess symptoms of insomnia, anxiety, depression, and posttraumatic stress symptoms (PTSS) in postoperative thyroid patients during the peak of the COVID-19 pandemic in China and to investigate factors associated with these psychological symptoms. The goal of our study was to report the mental health status of postoperative thyroid patients and provide a reference for conducting thyroid surgery and perioperative patient education during the COVID-19 outbreak based on the potential influencing factors.

## Materials and Methods

### Setting and Participants

From 2020.02.04 to 2020.02.18, when the COVID-19 epidemic was at its peak in China, we identified and contacted patients who had undergone thyroid surgery at Peking Union Medical College Hospital and were receiving regular follow-up in the outpatient clinic. An online questionnaire was sent to them *via* the contact information in the medical record system, and data were collected twice a day. The questionnaire was set to allow submission only after all questions were answered. According to the inclusion criteria, patients had to be 18-70 years of age, have undergone thyroid surgery, have had no surgical complications (e.g., permanent hypoparathyroidism, recurrent laryngeal nerve palsy, etc.) during hospitalization and follow-up, and can ensure the accuracy of the information. The presence of other malignancies or a history of diagnosed psychological disorders, either self-reported by patients or confirmed through the electronic medical records by the researchers, were used as exclusion criteria.

The study was approved by the Ethics Committee of Peking Union Medical College Hospital.

### Demographic and Clinical Characteristics

Patients’ sociodemographic characteristics, such as age, sex, employment status, marital status, education level, annual family income, and knowledge of their condition, were recorded from answers to the questionnaire, as well as whether their follow-up in the outpatient clinic which was usually once every 1 month to 3 months was disrupted by the COVID-19 pandemic and whether they searched for COVID-19-related news and medical information through social media. The clinical features of the patients, including comorbidities, time since surgery, type of surgery, and pathological results, were obtained by accessing the hospital’s electronic medical record system.

### Applied Questionnaires

Insomnia Severity Index (ISI): The 7-item ISI questionnaire is widely used to assess the symptoms and severity of insomnia (30); each question is scored from 0 to 4, for a total score of 28. Participants who scored 0-7 are classified as having no insomnia, 8-14 indicates mild insomnia, 15-21 indicates moderate insomnia and 22-28 indicates severe insomnia.

Generalized Anxiety Disorder Questionnaire (GAD-7): The GAD questionnaire consists of 7 questions measuring anxiety symptoms that are scored on a scale of 0 (“not at all”) to 3 (“nearly every day”) (31) for a total score of 21. A score of 0-4 indicates no anxiety, 5-9 indicates mild anxiety, 10-14 indicates moderate anxiety, and a score of more than 15 indicates severe anxiety.

Patient Health Questionnaire (PHQ-9): The PHQ is a questionnaire consisting of 9 items used to assess depression symptoms that are each scored from 0 (“not at all”) to 3 (“nearly every day”), for a total score of 27 (32). A score of 0-4 indicates no depression, 5-9 indicates mild depression, 10-14 indicates moderate depression, and a score of more than 15 indicates severe depression.

Impact of Events Scale-Revised (IES-R): The 22-item IES-R questionnaire is used to assess PTSS symptoms (33); each question is scored from 0 (“not at all”) to 4 (“extremely”), for a total score of 88. A score of 0-23 indicates no PTSS, 24-32 indicates mild PTSS, 33-36 indicates moderate PTSS and 37-88 indicates severe PTSS.

All questionnaires measured mental health symptoms within the past two weeks.

### Statistical Analysis

Categorical data are expressed as rates, and continuous data are expressed as the median (range) or mean (SD). Independent samples t-tests, one-way ANOVAs, and Kruskal-Wallis tests were used to analyze the association between the categorical variables and scores on each psychological symptom (i.e., insomnia, anxiety, depression, and PTSS), and Pearson correlation analyses were used to analyze the association between the continuous variables and psychological symptom scores. Independent variables identified as significant in the bivariate analysis were included in a multivariable linear regression model to analyze further the independent factors influencing each psychological symptom.

All analyses were conducted in SPSS (version 26.0; IBM) and GraphPad Prism (version 9.1.1), with a two-tailed p-value of ≤0.05 considered statistically significant.

## Results

### Patient Characteristics

Of the 248 postoperative patients who provided informed consent, seven were excluded: two could not ensure the accuracy of their information and five had a history of psychological disorders; thus, 241 patients were included in the study. The demographic and clinical characteristics of the patients are shown in **Table 1**. The median age of these patients was 4l years (range: 23-67 years), with 78.8% female (n=190), 72.2% employed or full-time students (n=174), 85.5% married (n=206), 76.8% possessing a college degree or higher education (n=185), 71.8% with an annual household income >60,000 RMB (n=173), and 81.7% in good general health with no comorbidities (n=197).

**Table 1:** Baseline characteristics of patients.

Characteristic	n (%)
Age, year [median (range)]	41 (23-67)
Sex	
Male	51 (21.1)
Female	190 (78.8)
Employment status	
Employed or full-time student	174 (72.2)
Unemployed	32 (13.3)
Retired	35 (14.5)
Marital status	
Married	206 (85.5)
Single, divorced, or widowed	35 (14.5)
Highest level of education	
High school or below	56 (23.2)
College or higher	185 (76.8)
Annual family income, RMB^1^	
≦60,000	68 (28.2)
>60,000	173 (71.8)
Number of comorbidities	
0	197 (81.7)
1	32 (13.3)
2	9 (3.7)
3	3 (1.2)
Time since surgery, month	
<6	78 (32.4)
6-11	89 (36.9)
≧12	74 (30.7)
Type of surgery	
Total thyroidectomy	170 (70.5)
Unilateral lobectomy	71 (29.5)
Pathology	
Benign	22 (9.1)
Malignant	219 (90.9)
Lymph node metastasis (N=219)	
Yes	91 (37.8)
No	128 (53.1)
Understand their condition	
Complete or basic understanding	228 (94.9)
Partial understanding	13 (5.4)
Self-identification of the severity	
Very serious or somewhat serious	94 (39.0)
Not too serious or not serious	147 (61.0)
Usual follow-up or treatment disrupted	
No	140 (58.1)
Yes	101 (41.9)
Social media information	
No	54 (22.4)
Yes	187 (77.6)

^1^ 1 RMB is equivalent to 0.16 USD.

Of the 241 patients, the majority underwent total thyroidectomy (70%, n=170) and had malignant pathology results (90.9%, n=219). The time since surgery was roughly evenly split, with 32.4%, 36.9%, and 30.7% of patients reporting that <6 months, 6-11 months, and >12 months had elapsed since surgery, respectively. The vast majority of patients felt that they had a complete or basic understanding of their condition (94.9%, n=228), and 61.0% (n=147) felt that their condition was not serious. During the peak period of the pandemic, 41.9% (n=101) of patients reported disruptions to their follow-up and treatment, and 77.6% of patients (n=187) used social media to search for news, medical information, and guidance related to COVID-19 while at home.

### Mental Health Outcomes

**Table 2** displays the results of the questionnaire corresponding to the 4 psychological symptoms, with median scores of 4 (IQR: 0-9) on the ISI, 3 (IQR: 0.5-7) on the GAD-7, 2 (IQR: 0-6.5) on the PHQ-9 and 10 (IQR: 2-22) on the IES-R, respectively. Of the 241 patients, those with symptoms of insomnia, anxiety, depression, and PTSS accounted for 32.0% (n=77), 39.4% (n=95), 33.6% (n=81), and 21.2% (n=51), respectively, with the majority of patients experiencing mild psychological symptoms and fewer experiencing moderate and severe symptoms; in contrast, there were more patients with severe PTSS than those with moderate PTSS according to the IES-R.

**Table 2:** Overview of insomnia, anxiety, depression and PTSS.

		n (241)	%
ISI	Median (IQR) 4 (0-9)		
	Normal	164	68.0
	Mild insomnia	61	25.3
	Moderate insomnia	14	5.8
	Severe insomnia	2	0.8
GAD-7	Median (IQR) 3(0.5-7)		
	Normal	146	60.6
	Mild anxiety	70	29.0
	Moderate anxiety	19	7.9
	Severe anxiety	6	2.5
PHQ-9	Median (IQR) 2 (0-6.5)		
	Normal	160	66.4
	Mild depression	56	23.2
	Moderate depression	15	6.2
	Severe depression	10	4.1
IES-R	Median (IQR) 10 (2-22)		
	Normal	190	78.8
	Mild PTSS	27	11.2
	Moderate PTSS	8	3.3
	Severe PTSS	16	6.6

ISI, Insomnia Severity Index; GAD-7, Generalized Anxiety Disorder Questionnaire; PHQ-9, Patient Health Questionnaire; IES-R, Impact of Events Scale-Revised.

### Factors Related to Mental Health

**Figure 1:**
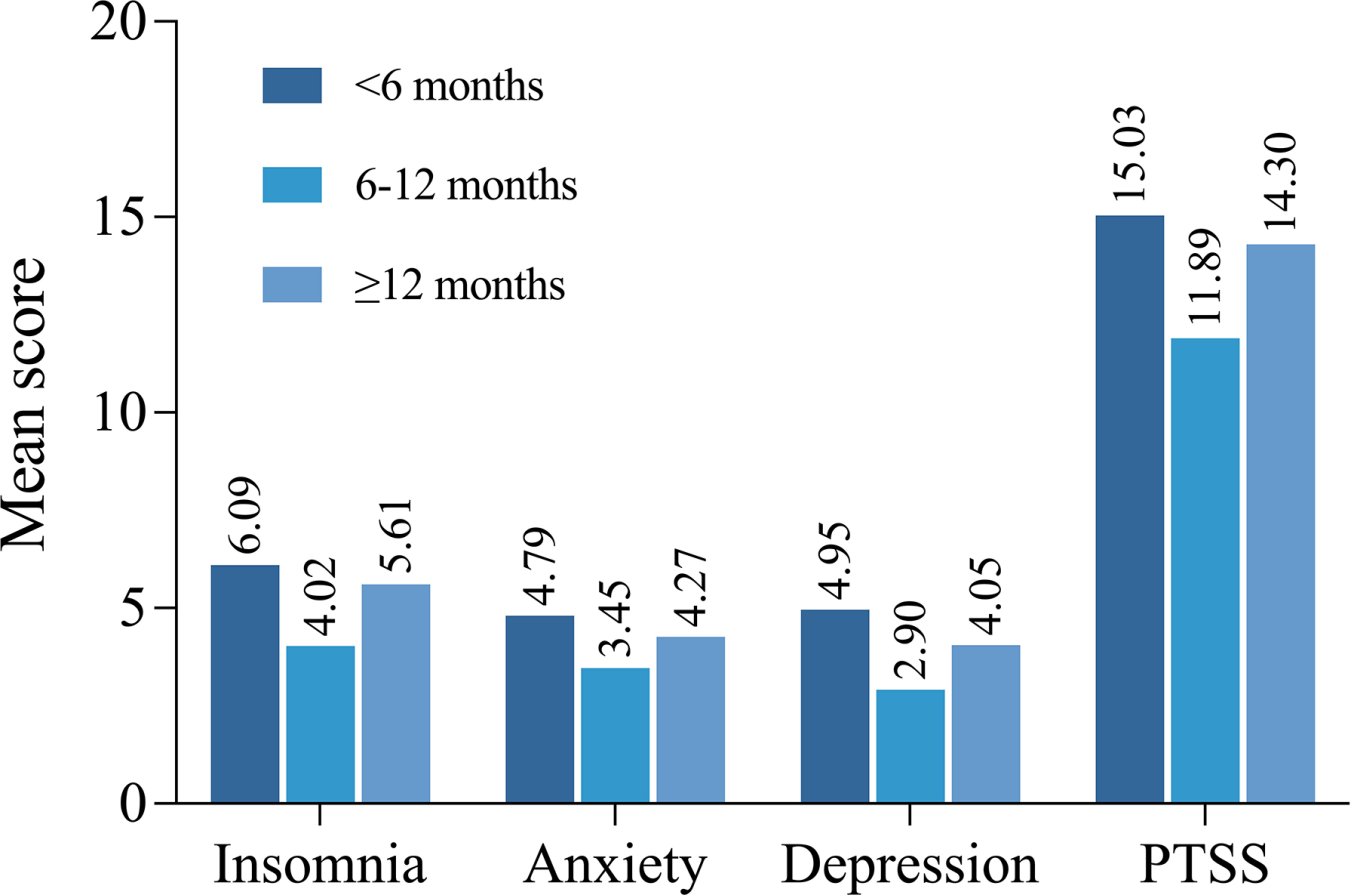
Comparison of the mean scores of insomnia, anxiety, depression and PTSS among patients at different time since surgery.

**Table 3** shows the association between demographic factors, clinical characteristics, and independent variables related to COVID-19 and mental health status. Bivariate analysis revealed that in postoperative thyroid patients, older patients had higher levels of PTSS during the peak period of COVID-19 (p=0.047), and single/divorced/widowed patients had higher levels of depression and PTSS than married patients (p=0.034 and p=0.022, respectively). Patients with a college degree or higher education had lower levels of insomnia, anxiety, and PTSS than patients with less-than-high school education (p=0.010, p=0.034, and p=0.012, respectively), and patients with a higher annual family income had lower levels of insomnia, depression, and PTSS (p=0.009, p=0.035, and p=0.036, respectively). The number of comorbidities was associated with every mental health dimension, and there was also a significant association between time since surgery and insomnia as well as depression. Surprisingly, patients that had surgery 6-11 months prior had the lowest levels of psychological symptoms of insomnia and depression, as shown in **Figure 1**. Patients who had undergone thyroid surgery within the past 6 months or more than 12 months ago had higher levels of insomnia and depression, and similar trends were found for anxiety and PTSS symptoms, although these were not significant. Insomnia and depression levels were significantly higher when patients knew less about their condition (p=0.022 and p=0.004, respectively). Patients whose routine follow-up was disrupted and those who searched social media for COVID-19-related information had higher levels of anxiety and PTSS than patients with non-disrupted follow-up (p=0.006 for GAD-7 scores, p=0.023 for IES-R scores) and those with less exposure to social media (p=0.008 for GAD-7 scores, p<0.001 for IES-R scores).

**Table 3:** Bivariate analysis of insomnia, anxiety, depression, and PTSS scores.

	ISI		GAD-7		PHQ-9		IES-R	
	Mean (SD)	*p*-value	Mean (SD)	*p*-value	Mean (SD)	*p*-value	Mean (SD)	*p*-value
Sex		0.689		0.355		0.707		0.266
Male	5.45 (5.91)		3.65 (3.46)		3.69 (4.55)		11.80 (12.44)	
Female	5.11 (5.35)		4.27 (4.44)		3.98 (5.04)		14.14 (13.49)	
Employment status		0.150		0.166		0.397		0.176
Employed or full-time student	4.78 (5.30)		3.82 (3.69)		3.67 (4.54)		12.81 (12.34)	
Unemployed	5.78 (5.54)		5.06 (5.44)		4.88 (5.49)		14.09 (13.32)	
Retired	6.63 (6.03)		4.89 (5.42)		4.29 (6.13)		17.37 (17.03)	
Marital status		0.089		0.061		**0.034**		**0.022**
Married	4.93 (5.37)		3.87 (4.00)		3.56 (4.59)		12.84 (13.14)	
Single, divorced, or widowed	6.63 (5.81)		5.69 (5.32)		6.00 (6.30)		18.37 (13.32)	
Highest level of education		**0.010**		**0.034**		0.110		**0.012**
High school or below	6.82 (5.21)		5.36 (5.06)		4.84 (5.02)		17.93 (14.66)	
College or higher	4.68 (5.45)		3.77 (3.91)		3.64 (4.88)		12.35 (12.59)	
Annual family income, RMB^1^		**0.009**		0.099		**0.035**		**0.036**
≦60,000	6.63 (5.99)		4.97 (5.25)		5.18 (6.22)		16.50 (15.08)	
>60,000	4.61 (5.15)		3.81 (3.75)		3.42 (4.24)		12.52 (12.37)	
Number of comorbidities		**0.012**		**0.002**		**0.009**		**0.007**
0	5.23 (5.46)		4.29 (4.41)		3.97 (5.03)		13.62 (13.49)	
1	3.53 (5.23)		2.09 (2.13)		2.38 (3.85)		9.69 (10.32)	
2	8.00 (4.85)		6.33 (2.74)		6.56 (3.40)		24.11 (9.56)	
3	10.67 (3.06)		9.33 (5.69)		9.00 (7.00)		26.00 (18.68)	
Time since surgery, month		**0.010**		0.055		**0.017**		0.202
<6	6.09 (5.90)		4.79 (4.42)		4.95 (5.36)		15.03 (13.46)	
6-11	4.02 (5.19)		3.45 (4.18)		2.90 (4.57)		11.89 (12.89)	
≧12	5.61 (5.13)		4.27 (4.10)		4.05 (4.69)		14.30 (13.50)	
Type of surgery		0.674		0.299		0.231		0.846
Total thyroidectomy	5.08 (5.35)		3.95 (4.13)		3.67 (4.75)		13.54 (12.92)	
Unilateral lobectomy	5.41 (5.76)		4.58 (4.52)		4.51 (5.32)		13.90 (14.19)	
Pathology		0.651		0.794		0.815		0.935
Benign	5.68 (5.54)		4.36 (4.87)		3.68 (4.50)		13.86 (14.58)	
Malignant	5.13 (5.47)		4.11 (4.20)		3.94 (4.98)		13.62 (13.18)	
Lymph node metastasis (N=219)		0.815		0.779		0.987		0.392
Yes	5.23 (5.80)		4.21 (4.63)		3.93 (5.41)		14.53 (14.58)	
No	5.05 (5.24)		4.05 (3.88)		3.95 (4.67)		12.98 (12.10)	
Understand their condition		**0.022**		0.175		**0.004**		0.083
Complete or basic understanding	4.99 (5.33)		4.05 (4.25)		3.70 (4.77)		13.29 (13.05)	
Partial understanding	8.54 (6.91)		5.69 (4.15)		7.69 (6.20)		19.85 (16.19)	
Self-identification of the severity		0.576		0.350		0.491		0.602
Very serious or somewhat serious	5.43 (5.58)		4.46 (4.68)		4.19 (5.41)		14.20 (13.74)	
Not too serious or not serious	5.02 (5.41)		3.93 (3.96)		3.74 (4.61)		13.29 (13.01)	
Usual follow-up or treatment disrupted		0.056		**0.006**		0.323		**0.023**
No	4.61 (5.12)		3.50 (4.00)		3.65 (5.03)		11.99 (12.47)	
Yes	5.97 (5.84)		5.02 (4.44)		4.29 (4.79)		15.93 (14.07)	
Social media information		0.145		**0.008**		0.340		**<0.001**
No	4.22 (5.52)		2.80 (3.91)		3.35 (5.34)		7.91 (10.85)	
Yes	5.45 (5.42)		4.52 (4.28)		4.08 (4.81)		15.30 (13.48)	
	r		r		r		r	
Age, year	0.099	0.126	0.107	**0.099**	0.006	0.930	0.128	**0.047**

^1^ 1 RMB is equivalent to 0.16 USD

The bold entries indicate variables with p value less than 0.05.

**Figure 2:**
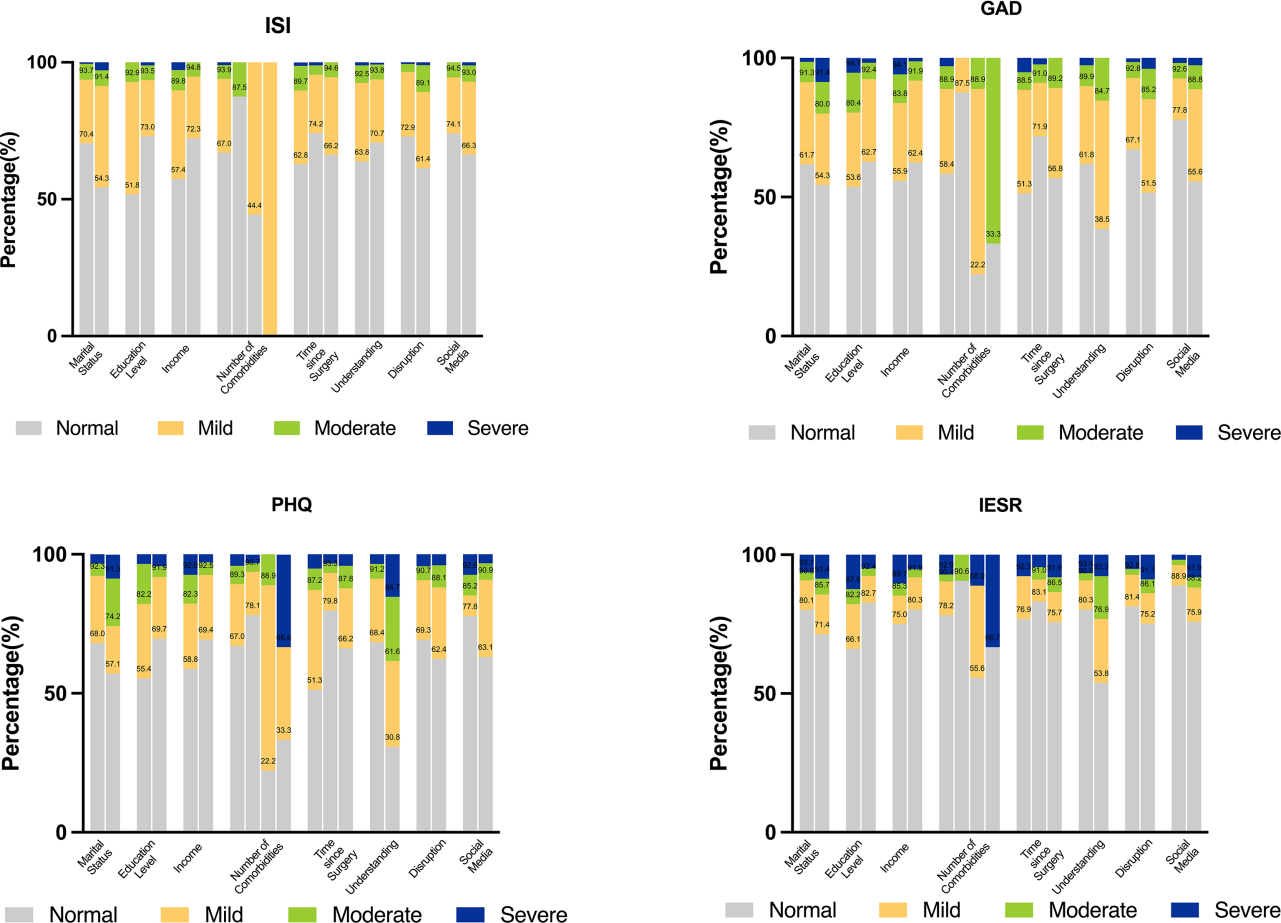
Stacked bar blots to identify the distribution of baseline characteristics in distinct degrees of psychological symptom. (Interpretations: Marital status: Married vs. Single/divorced/widowed; Education level: High school or below vs. College or higher; Income: ≦60,000 vs.>60,000; Number of comorbidities: 0 vs. 1 vs. 2 vs. 3; Time since surgery: <6 vs. 6-11 vs. ≧12; Understanding: Complete or basic vs. Partial; Disruption: No vs. Yes; Social Media: No vs. Yes).

**Figure 3:**
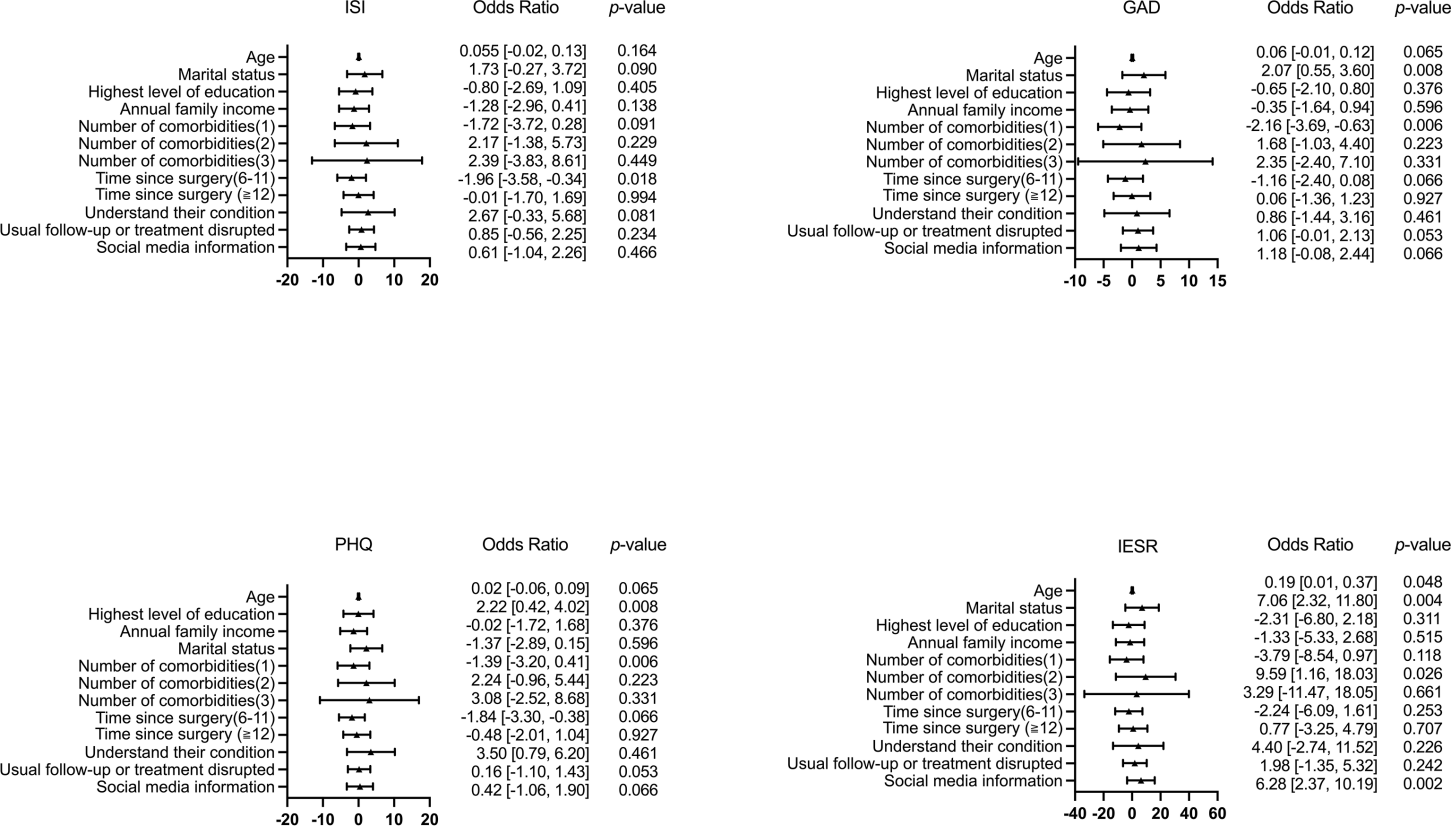
Forest plots to identify independent affecting factors for insomnia, anxiety, depression, and PTSS.

Based on the results of the bivariate analysis, nine variables – age, marital status, highest level of education, annual family income, number of comorbidities, time since surgery, understanding their condition, usual follow-up or treatment disrupted, and social media information – were included in the multivariate linear regression model. **Figure 2** shows the distribution of baseline characteristics of these variables in distinct degrees of psychological symptoms. The results of the multivariable linear regression are presented in **Table 4**. Time since surgery was an independent factor related to insomnia [odds ratios (ORs), -1.96 (95% CI, -3.58 to -0.34)]; marital status [ORs, 2.07 (95% CI, 0.55 to 3.60)] and number of comorbidities [ORs, -2.16 (95% CI, -3.69 to -0.63)] were independent factors related to anxiety; marital status [ORs, 2.22 (95% CI, 0.42 to 4,02)], time since surgery [ORs, -1.84 (95% CI, -3.30 to -0.38)], and knowledge of their condition [ORs, 3.50 (95% CI, 0.79 to 6.20)] were independent factors affecting depression; and age [ORs, 0.19 (95% CI, 0.01 to 0.37)], marital status [ORs, 7.06 (95% CI, 2.32 to 11.80)], number of comorbidities [ORs, 9.59 (95% CI, 1.16 to 18.03)] and use of social media for COVID-19 information [ORs, 6.28 (95% CI, 2.37 to 10.19)] were independent factors influencing PTSS. The degree of influence of these factors were displayed in **Figure 3**.

**Table 4:** Multivariable linear regression analysis of insomnia, anxiety, depression and PTSS scores.

	ISI	GAD-7	PHQ-9	IES-R
	OR [95%CI]	OR [95%CI]	OR [95%CI]	OR [95%CI]
Age	0.055 [-0.02, 0.13]	0.06 [-0.01, 0.12]	0.02 [-0.06, 0.09]	**0.19 [0.01, 0.37]**
Marital status				
Married				
Single, divorced, or widowed	1.73 [-0.27, 3.72]	**2.07 [0.55, 3.60]**	**2.22 [0.42, 4.02]**	**7.06 [2.32, 11.80]**
Highest level of education				
High school or below				
College or higher	-0.80 [-2.69, 1.09]	-0.65 [-2.10, 0.80]	-0.02 [-1.72, 1.68]	-2.31 [-6.80, 2.18]
Annual family income, RMB				
≦60,000				
>60,000	-1.28 [-2.96, 0.41]	-0.35 [-1.64, 0.94]	-1.37 [-2.89, 0.15]	-1.33 [-5.33, 2.68]
Number of comorbidities				
0				
1	-1.72 [-3.72, 0.28]	**-2.16 [-3.69, -0.63]**	-1.39 [-3.20, 0.41]	-3.79 [-8.54, 0.97]
2	2.17 [-1.38, 5.73]	1.68 [-1.03, 4.40]	2.24 [-0.96, 5.44]	**9.59 [1.16, 18.03]**
3	2.39 [-3.83, 8.61]	2.35 [-2.40, 7.10]	3.08 [-2.52, 8.68]	3.29 [-11.47, 18.05]
Time since surgery, month				
<6	ref	ref	ref	ref
6-11	**-1.96 [-3.58, -0.34]**	-1.16 [-2.40, 0.08]	**-1.84 [-3.30, -0.38]**	-2.24 [-6.09, 1.61]
≧12	-0.01 [-1.70, 1.69]	0.06 [-1.36, 1.23]	-0.48 [-2.01, 1.04]	0.77 [-3.25, 4.79]
Understand their condition				
Complete or basic understanding	ref	ref	ref	ref
Partial understanding	2.67 [-0.33, 5.68]	0.86 [-1.44, 3.16]	**3.50 [0.79, 6.20]**	4.40 [-2.74, 11.52]
Usual follow-up or treatment disrupted				
No	ref	ref	ref	ref
Yes	0.85 [-0.56, 2.25]	1.06 [-0.01, 2.13]	0.16 [-1.10, 1.43]	1.98 [-1.35, 5.32]
Social media information				
No	ref	ref	ref	ref
Yes	0.61 [-1.04, 2.26]	1.18 [-0.08, 2.44]	0.42 [-1.06, 1.90]	**6.28 [2.37, 10.19]**

The bold entries indicate variables with p value less than 0.05.

## Discussion

The psychological and emotional issues in numerous populations during the COVID-19 pandemic have received increasing attention (11–21). While psychological symptoms such as insomnia, anxiety, depression, and reduced quality of life after thyroid surgery have been reported by patients (34–36), the mental health of postoperative thyroid patients during the pandemic and the factors affecting it remain relatively unknown (37). To our knowledge, this current study is the first investigating mental health and influencing factors during the peak of the COVID-19 pandemic.

Our study found that a significant proportion of postoperative thyroid patients experienced insomnia, anxiety, depression, and PTSS, mostly to a mild degree. Age, marital status, education level, annual family income, number of comorbidities, time since surgery, the patients’ knowledge of their condition, disruption to follow-up during the COVID-19 pandemic, and coping strategies were associated with one or more of the psychological symptoms of insomnia, anxiety, depression, and PTSS.

Overall, we found that during the COVID-19 pandemic, postoperative thyroid patients reported more symptoms of insomnia, anxiety, depression, and PTSS than the normal population and worse psychological status than postoperative thyroid patients before COVID-19 (1, 11, 36); however, these patients had better mental health than postoperative patients with other types of cancers (18).

Consistent with previous studies, we found that single/divorced/widowed patients were at a higher risk of psychological symptoms than married since they received more emotional support (36, 38). Higher education levels and higher annual family income were associated with lower psychological symptoms, consistent with the literature (11, 36), possibly because patients with higher education levels usually have more comprehensive information about both their condition and the COVID-19 pandemic. Moreover, higher income suggests higher risk tolerance. Together, these factors may support patients in coping with negative emotions and prevent the development of psychological disorders.

As shown in **Table 1**, almost a third of the patients underwent thyroid surgery more than 12 months before enrollment, and we set time since surgery as a variable for analysis. Notably, we found that it was an independent factor related to insomnia and depression and had nonsignificant but similar associations with anxiety and PTSS scores. Interestingly, patients who had undergone surgery within the past 6 months reported the most severe psychological symptoms, followed by those who underwent surgery more than 12 months ago, while patients who had surgery between 6 and 12 months ago had the best mental health. Chen et al. have shown that symptoms of anxiety and depression in patients become milder within 1 year after surgery (39), similar to our results. A possible explanation is that patients’ lives gradually return to normal after surgery. However, our results are novel in that patient mental health symptoms worsened again 1 year after surgery; this unanticipated result could stem from an increase in fear of recurrence after 1 year had elapsed (40–42).

According to the multivariable linear regression model, we found a significant association between the number of comorbidities and all 4 mental health symptoms: having 2 comorbidities was an independent risk factor for PTSS. Surprisingly, however, patients with one comorbidity reported the lowest scores on the psychological scales, i.e., had better mental health than that patients without comorbidities. This result has not been previously reported and is contrary to our expectations; therefore, it should be treated with caution. It is difficult to explain this observation, but it might be related to fatigue due to comorbidity (43). Another possible explanation is that patients with one comorbidity tend to be more conscious and well informed about their physical health and therefore experienced fewer mood fluctuations due to the COVID-19 pandemic than patients without comorbidities, whereas the poor mental health of patients with two or more comorbidities may be due to their poorer general condition (44).

Another interesting finding was that proactive search of COVID-19-related information and medical guidance from social media was associated with higher levels of anxiety and PTSS and was an independent risk factor for PTSS. This is consistent with the findings of previous studies (45–48). Potentially, information overload on social media makes it difficult for people with insufficient expertise to distinguish between truths and falsehoods. Especially for patients who are worried about their condition and those who cannot visit hospitals due to the pandemic, information overload increases their anxiety and PTSS about COVID-19 (17, 45, 49).

Our study is the first to report on the mental health of postoperative thyroid patients during the peak of the COVID-19 pandemic and the potential factors that influenced mental health. However, some possible limitations should be noted. First, we did not enroll a control group, although we made comparisons to previous studies. Second, this was a single-center study, and our patients were all from nearby areas; the hospital was far from Wuhan, the center of the epidemic in China. In addition, there was no specific preoperative mental assessment to determine the potential impact of thyroid function on their psychological status, which may influence postoperative mental health.

According to our findings, patients who were older, single/divorced/widowed, less educated, had lower annual income, in poor general health, had undergone surgery within the past six months, had disrupted access to postoperative follow-up, and actively searched social media for COVID-19-related information were more likely to develop mental health problems. These findings suggest that older, single/divorced/widowed patients should be encouraged to reach out more to friends and seek emotional support. Additionally, patients with lower levels of education, lower income, more comorbidities, and those who have undergone surgery within the past 6 months are at higher risk of developing mental health problems and need to be promptly identified to provide more psychological support and intervention.

In addition, the mental health of patients might be improved by enhancing patient education and improving their understanding of their condition. Providing postoperative patients with medical care during the COVID-19 pandemic, such as educating patients to correctly identify medical information on social media, providing psychological counseling services for patients during special periods, offering special online consultation section, and organizing inter-patient support to alleviate patients’ anxiety and PTSS.

## Data Availability Statement

The raw data supporting the conclusions of this article will be made available by the authors, without undue reservation.

## Ethics Statement

The studies involving human participants were reviewed and approved by the Ethics Committee of Peking Union Medical College Hospital. The patients/participants provided their written informed consent to participate in this study. Written informed consent was obtained from the individual(s) for the publication of any potentially identifiable images or data included in this article.

## Author Contributions

SY data analysis, data interpretation, original manuscript drafting; XX: study concept and design, data collection, data interpretation, original manuscript drafting and editing. All authors contributed to the article and approved the submitted version.

## Funding

This study is supported by the National Natural Science Foundation of China (32071436) and the Beijing Municipal Natural Science Foundation (7222127).

## Conflict of Interest

The authors declare that the research was conducted in the absence of any commercial or financial relationships that could be construed as a potential conflict of interest.

## Publisher’s Note

All claims expressed in this article are solely those of the authors and do not necessarily represent those of their affiliated organizations, or those of the publisher, the editors and the reviewers. Any product that may be evaluated in this article, or claim that may be made by its manufacturer, is not guaranteed or endorsed by the publisher.
